# A New Nucleolar Body Appears in *Drosophila saltans* Salivary Gland Cells Before Histolysis, in Programmed Cell Death

**DOI:** 10.1673/031.006.4401

**Published:** 2006-12-05

**Authors:** C. C. de Oliveira, H. E. M. de Campos Bicudo

**Affiliations:** ^1^ Program in Ecology and Evolutionary Biology, Department of Biological Sciences, University of Arkansas, Fayetteville, AR 72701, USA; ^2^ Universidade Estadual Paulista - UNESP, Departamento de Biologia, Rua Cristovão Colombo, 2265, 15054-000 São José do Rio Preto, SP, Brazil

**Keywords:** salivary gland, nucleolar and chromosome changes in cell death, *Drosophila saltans*

## Abstract

The salivary glands of *Drosophila saltans* (*saltans* group, *saltans* subgroup) analyzed in an advanced stage of programmed cell death showed the appearance of a single, round, nucleolar corpuscle inside the highly altered nucleus of every gland cell, at a time during which the integrity of the original nucleolus was already lost and the original nucleolar material apparently disappeared. In the same nuclei, which already had also lost the characteristic chromosome structure, some delicate chromosome threads were maintained. In many cells, the new nucleolar corpuscle and these chromosome threads are associated. These findings are novel. However, the hypothesis put forward concerning their meaning remains dependent on other studies.

## Introduction

Programmed cell death is an essential phenomenon in normal development and adulthood of multicellular organisms (Abrams et al. 1993; Rusconi et al. 2000; Bursch 2001). In insect development, it is involved in tissue reorganization that establishes the characteristics of the adult body. Such reorganization requires the elimination of several tissues including the salivary glands in *Drosophila*. In this organism, as in other insects, the histolysis of the salivary glands due to programmed cell death is controlled by the steroid hormone ecdysone the levels of which regulate gene expression spatially, in both inductive and repressive ways, causing the elimination of the glands (Rusconi et al. 2000).

The caspases proteases play an essential role in the programmed cell death of *Drosophila* salivary glands (Lee et al. 2003; Martin and Baehrecke 2003). Caspases form an enzyme cascade that can be separated into activators, effectors and negative regulators. The expression of the effector genes such as *reaper*, *hid* and *dronc* is upregulated just prior to the onset of death while the expression of the cell death inhibitor *diap2* is repressed (Jiang et al. 1997; Dorstyn et al. 1999; Lee and Baehrecke 2001). These, and other effector genes, seem to be under control of *E93*, an ecdysone-induced primary response gene (Lee et al. 2000; Gorski and Marra 2002). Preceding salivary gland histolysis, the levels and localization of filamentous actin, α-tubulin, α-spectrin and nuclear lamins change, parallel to an increase in the levels of active caspase 3 and a cleaved form of nuclear lamin (Martin and Baehrecke 2003). More recently, noncoding micro RNAs involved in the regulation of cell death were described (Xu et al. 2003; 2004).

Morphological studies of the programmed cell death of *Drosophila* salivary glands are mostly related to the study of whole salivary glands examined using nuclear staining with acridine orange and the TUNEL assay in order to determine whether salivary gland cell death is accompanied by DNA fragmentation, which is considered indicative of apoptosis. The cells examined in the whole glands are described as characterized by “pycnotic nuclei, breakdown of the nuclear membranes and rupture of the basement membranes covering the gland cells” (Jiang et al. 1997). In relation to the nucleolus, some authors already recognized that, in general, little attention has been focused on this organoid during apoptosis, considering it as an area of neglected opportunities in research of this process (Kerr 1995; Reipert et al. 1999).

In the present study, instead of using whole glands the changes in the polytene chromosomes and the nucleolus during programmed cell death were observed in *Drosophila saltans* salivary gland squash preparations using labeling techniques for light microscopy that are recognized as markers for nuclear components. The appearance of a single, round, nucleolar corpuscle inside the highly altered nucleus of every degenerationg gland cell is described.

## Materials and Methods

### The species and developmental stages used

*D. saltans* (*saltans* group, *saltans* subgroup) from San José, Costa Rica (strain 180.40) was used. This strain has been maintained in the laboratory in a corn starch-agar culture medium, at a temperature of 20°C ± 1°C. Developmental stages used to analyze salivary gland structures were: late third instar larvae, prepupae, young pupae and intermediate pupae, aged a mean of 250, 251, 278 and 338 hours from the egg, respectively.

### Microscopy preparations and staining

Larvae and pupae were dissected in Demerec solution (Demerec and Kaufmann 1945). The salivary glands were fixed for one minute in 45% acetic acid and squashed in the same solution. After squashing, the preparations were frozen in liquid nitrogen and the coverslips removed. The slides were dried at room temperature and kept in the freezer for later staining. This procedure was used for slides that were stained by silver nitrate (Howell and Black 1980), Feulgen reaction (Kirschbaum 1973) and acridine orange (adapted to salivary glands from Abrams et al. 1993).

In acridine orange staining, squashed salivary glands were incubated for 15 minutes in 5 µg/ml acridine orange (Sigma, www.sigmaaldrich.com) in 0.1 M phosphate buffer, pH 7.2. A coverslip was placed on the material and the analysis was performed using fluorescence microscopy (Zeiss Axioskop-MC 80, www.zeiss.com) and filters for blue, pink and red fluorescence.

For lacto-acetic orcein stained preparations, the salivary glands were placed in a drop of 2% orcein on the slide for eight minutes, then transferred to a drop of 50% lactic acid for 10 minutes and squashed (adapted from Bicudo 1981).

The fluorescent stain, acridine orange, has been used to identify apoptotic cells in *Drosophila* embryos (Abrams et al. 1993; White et al. 1994). Acridine orange is a metachromatic dye in fixed tissues, staining single-stranded nucleic acids orange and double-stranded nucleic acids green. Lacto-acetic orcein technique, which with some variations has been used for more than half a century in chromosome analysis, stains total proteins bound to DNA, enabling the chromatin to be observed (Ashburner 1989). The Feulgen reaction is specific for DNA staining (Feulgen and Rossenbeck 1924; Melo and Vidai 1978). The silver nitrate technique (Goodpasture and Bloom 1975) with some variations, has been intensively used as a marker for nucleolar organizing regions and nucleoli, in normal and in pathological tissues. Several proteins required for rRNA transcription and preribosome processing have the ability to reduce silver nitrate, staining the nucleolus strong brown. Most silver-positive nucleolar proteins adhere to the nucleolar organizing regions during mitosis, hence they are named Ag-NOR proteins (Scwarzacher and Wachtter 1993). The validity of this procedure is reinforced by the fact that it has been applied to eliminate false-negative results from whole-mount immunocytochemistry due to insufficient individual antigen concentration (Bernard et al. 2001).

### Photomicrographs

All fluorescent micrographs were taken with an automatic camera loaded with Kodak Ultra 400 ASA film. For micrographs taken from slides prepared with the other stain techniques, Kodak TMAX black and white 100 ASA film was used, in a Zeiss II Photomicroscope.

### Results and Discussion

In *D. saltans*, salivary gland histolysis occured during the intermediate pupal stage, aged a mean of 338 hours from the egg (minimum 269 and maximum 406 hours) as observed in the conditions of the present experiment. The entire process, from the beginning of chromosome changes in the prepupae to histolysis, lasted a mean of 80 hours. The nuclear alterations did not occur at the same time in all cells of a salivary gland, and the alterations did not simultaneously affect all the chromosomes in a cell, not even every chromosome entirely, suggesting that some chromosome regions remained active for a longer time. In addition, we found a single salivary gland of the pair in about 10% of intermediate pupae dissected for slide preparations, suggesting that histolysis of the two members of the pair may also be asynchronous. Berendes and Ashburner (1978) also observed that the two glands might differ in the timing of onset of histolysis.

**Figure 1.**
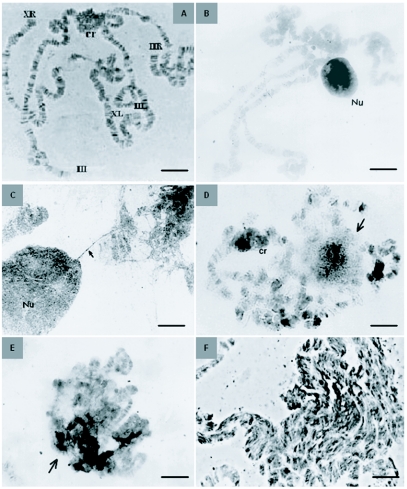
A-F. Salivary gland cells in different stages of the programmed cell death. A. Normal chromosome set (three chromosome pairs) in a third instar, lacto-acetic orcein stained larva cell. B-E. AG-stained cells. B. Normal nucleolus in third instar larva. C. Normal nucleolus associated with the nucleolar organizer region by DNA threads (arrow). D, E. Patterns of Ag-staining of the nucleolus (arrows) in different moments of programmed cell death: D. staining restrict to the nucleolar center; E. The nucleolus already lost its round shape, masses of stained nucleolar material being spread over and between masses of chromosome material. F. Phase contrast, lacto-acetic orcein. The chromosomes in advanced stage of structural change. Cr = chromocenter; Nu = nucleolus. Scale bars: A = 10 µm; B = 2.5 µm; C = 4.7 µm, D = 7.0 µm; E, F = 3.2 µm.

*D. saltans* has three polytene chromosome pairs, two metacentric-chromosomes X and II, and one acrocentric-chromosome III, all of them linked by the centromeric region forming the chromocenter (Figure 1A). As in most *Drosophila* species in the late third instar, the characteristic transverse pattern of bands and interbands along the length of the chromosomes was well defined, except in the heterochromatic centromeric region. At this developmental stage each salivary gland cell showed a large, single, round or oval nucleolus that stained strongly brown with silver nitrate and had a heterogeneous structure, as described using light and electron microscopy in several organisms (e.g., Bernhard 1966; Thiry and Goessens 1996). Chromatin threads linked the nucleolus to the nucleolar organizing region, which in *D. saltans* was located in the centromeric region of the X chromosome (Bicudo 1973) (Figure 1B, C). In *Drosophila*, these chromatin threads that are associated with the nucleolus stain brown with silver nitrate and are marked with rRNA by *in situ* hybridization (Bicudo 1981; Bicudo and Richardson 1981).

**Figure 2.**
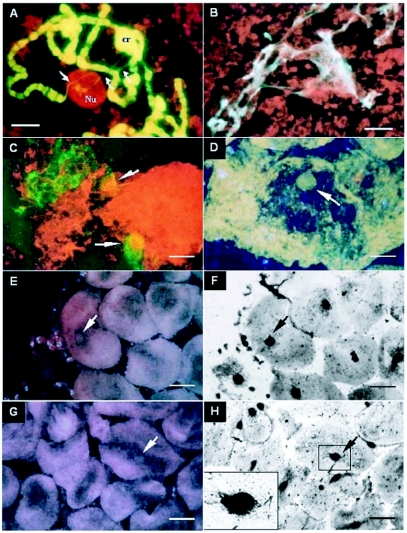
A-H. Salivary gland cells in different stages of programmed cell death. A. Orange-stained nucleolus (Nu) and green-stained chromosomes in a third instar larva cell stained with acridine orange technique. B. Advanced stage of chromosome alteration; the chromosomes remain green with acridine orange technique. C, D. Cells in very advanced stage of programmed cell death: the new nucleolar body (arrows) is associated with green-stained remains of chromosome material in two disrupted cells (C), or associated with the nuclear membrane (D). E-H. Cells also in very advanced stage of programmed cell death, stained with acridine orange (E, G) and with Ag-Nor (F, H). The arrows point to the same nucleolar bodies in slides stained with the two techniques. In H, the highlighted nucleolus is associated with fine, silver stained threads. In C to H, the cytoplasmic material looks very dense. Scale bars: A = 5.0 µm; B, C, D = 7.2 µm; E-H = 11.5 µm.

In acridine orange stained slides of late third instar larvae, the chromosomes were green and the nucleolus was orange or red, as expected on the basis of the properties of the stain. The chromatin associated with the nucleolus was well marked, as well as the nucleolus connection with the chromocenter region (Figure 2A). As development progresses, gradual loss of the chromosome-banding pattern, loss of the chromosome individuality, and transformation of the chromosomes into tenuous masses of filaments or tangled threads were observed (Figures 1F, 2B). The nucleolar integrity was also lost in parallel with the loss of its characteristic pattern of silver nitrate impregnation that either became restricted to inner regions of the nucleolus or became irregular (Figure1D, E), unlike the larval stage in which impregnation reaches the whole nucleolar mass. The nucleolar alterations increased until a differentiated nucleolar structure is no longer visible.

It is interesting that, in the last steps before histolysis, some remains of chromosome threads were still observed inside the nucleus that seems partially “empty” in cells stained with acridine orange (Figure 2C). These chromosome threads also stained with orcein and Feulgen, showing that DNA was present in them. In cells stained with acridine orange, they retained the green color observed in the intact chromosomes of larvae, and this staining pattern is different from that described in the literature, according to which the DNA in apoptotic chromosomes undergoes breakage that changes its acridine orange staining from the characteristic green to red (Abrams et al. 1993; Mpoke and Wolfe 1997; Nicoletti and Mannucci 2002; Berg 2002).

Just before histolysis, the chromosome threads appeared to be connected to a rounded corpuscle, now visible inside all salivary gland cell nuclei, that appears to be the single visible structure inside the acridine orange-stained cells (Figure 2D-H). Even when present outside the nucleus, in broken cells, the corpuscle maintained its integrity, indicating consistence. In the disrupted, or not disrupted nucleus, it was seen frequently close to, or associated with, the nuclear membrane (Figure 2D). Some of the acridine orange -stained preparations were stained using the silver nitrate technique, which stained the corpuscle strongly brown, confirming its nucleolar nature (Figure 2F, H). The fine chromosome threads connected with the corpuscle also stained brown with the silver nitrate, suggesting their involvement in nucleolar synthesis (Figure 2H).

The maintenance of chromosomal material and the appearance of a new nucleolar body at this advanced stage of programmed cell death in the salivary glands are remarkable. However, any explanation of these observations remains conjectural. Its presence may be indicative of new synthesis of enzymes for the last steps of salivary gland histolysis. In this case, the capability of protein synthesis would still subsist in the cytoplasm, when cell degradation is advanced and the polytene chromosomes are reduced to those few threads frequently associated with the nucleolar body. The previously mentioned reports of the involvement of microRNAs in the control of cell death in *Drosophila* suggests that noncoding rRNA synthesis is another possibility for the present findings. Other studies are necessary to evaluate these hypotheses.
